# Anti-Inflammatory Effects of GM1 Ganglioside on Endotoxin-Induced Uveitis in Rats

**DOI:** 10.3390/biom12050727

**Published:** 2022-05-21

**Authors:** Tzu-Heng Weng, Chang-Chih Ke, Yuahn-Sieh Huang

**Affiliations:** 1 Department of Ophthalmology, Tri-Service General Hospital, School of Medicine, National Defense Medical Center, Taipei 11490, Taiwan; aheng7435@gmail.com (T.-H.W.); frankke.tw@gmail.com (C.-C.K.); 2 Graduate Institute of Medical Sciences, National Defense Medical Center, Taipei 11490, Taiwan; 3 Graduate Institute of Aerospace and Undersea Medicine, National Defense Medical Center, Taipei 11490, Taiwan; 4 Department of Biology and Anatomy, National Defense Medical Center, Taipei 11490, Taiwan

**Keywords:** GM1 ganglioside, endotoxin-induced uveitis, iris-ciliary body, anti-inflammation, RAW 264.7 cells

## Abstract

Exogenous ganglioside GM1 has been reported to exert an immunomodulatory effect. We investigated the anti-inflammatory effect of GM1 ganglioside on endotoxin-induced uveitis (EIU) in rats and RAW 264.7 macrophages. Methods: EIU was induced in Lewis rats by administering a subcutaneous injection of lipopolysaccharide (LPS). GM1 was injected intraperitoneally for three consecutive days prior to the LPS injection. Twenty-four hours after the LPS injection, the integrity of the blood-aqueous barrier was evaluated by determining the protein concentration and number of infiltrating cells in the aqueous humor (AqH). Immunohistochemical and Western blot analyses of the iris-ciliary body (ICB) were performed to evaluate the effect of GM1 on the LPS-induced expression of cyclooxygenase-2 (COX-2) and intercellular adhesion molecule-1 (ICAM-1). The effect of GM1 on proinflammatory mediators and signaling cascades was examined in LPS-stimulated RAW 264.7 cells using Western blotting and immunofluorescence staining to further clarify the underlying anti-inflammatory mechanism. Results: GM1 significantly reduced the protein concentration and number of infiltrating cells in the AqH of rats with EIU. GM1 also decreased the LPS-induced expression of the ICAM-1 and COX-2 proteins in the ICB. In RAW 264.7 cells, GM1 inhibited the proinflammatory mediators induced by LPS, including inducible nitric oxide synthase (iNOS), COX-2, tumor necrosis factor-α (TNF-α), interleukin-1β (IL-1β) and interleukin-6 (IL-6), and this inhibitory effect was potentially mediated by suppressing transforming growth factor-β-activated kinase 1 (TAK1) and reactive oxygen species (ROS)-mediated activation of nuclear factor-κB (NF-κB) and mitogen-activated protein kinases (MAPKs). Conclusions: Based on this study, GM1 may be a potential anti-inflammatory agent for ocular inflammatory diseases.

## 1. Introduction

Among all ocular inflammatory diseases, uveitis is a serious vision-threatening disease and one of the leading causes of vision loss [1]. It mainly affects the uvea but may also affect adjacent structures such as the retina and vitreous [2,3]. Although autoimmune diseases, infections, and toxins are presumed to be the cause of the disease, the detailed pathogenesis remains unclear [3]. Endotoxin-induced uveitis (EIU) is a well-established animal model that can be induced by footpad injection in rodents and produces an acute form of uveitis following exposure to endotoxins such as lipopolysaccharide (LPS), which is a potent stimulator of the inflammatory response [4,5]. EIU represents a reliable experimental model to study the pathological mechanisms of uveitis and to assess the pharmacological efficacy of potential drugs for inhibiting inflammation [5]. The pathology of EIU is characterized by intense and acute but transient infiltration of the anterior chamber and vitreous cavity by neutrophils and macrophages without significant systemic symptoms except temporal diarrhea, mimicking the pathological progression of acute uveitis in humans [6,7,8,9]. The acute inflammatory response for EIU occurs 4 h after LPS injection, peaks at 18 to 24 h, and is maintained for 72 h [6,10]. LPS engages Toll-like receptor 4 (TLR4) and induces the release of critical proinflammatory factors such as nitric oxide (NO), prostaglandin (PG)-E2, tumor necrosis factor-α (TNF-α), interleukin (IL)-1β and IL-6 [11]. Ultimately, the accumulation of proinflammatory factors leads to disruption of the blood-ocular barrier and infiltration of inflammatory cells into both anterior and posterior segments of the eye, which contributes to the pathological progression of EIU [12,13]. Currently, topical systemic administration of corticosteroids remains the standard treatment for uveitis [14]. However, this treatment causes many undesirable ocular adverse effects [14,15]. Therefore, studies of the mechanism of intraocular inflammation and the development of effective and safe drugs remain important issues.

Ganglioside is a complex structural molecule composed of a glycosphingolipid (ceramide and oligosaccharide) with one or more sialic acids. It is a component of cell membranes in mammals and some lower animals [16,17,18] and is located in the lipid rafts of cell membranes [19]. Accumulating evidence indicates that gangliosides are abundant in the nervous system and are associated with the maintenance and repair of neural tissues [20,21]. They also participate in various cellular processes, such as cell growth, differentiation, intercellular interactions, adhesion, migration and apoptosis [22,23]. Ganglioside deficiency leads to damage to nerve tissues and inflammatory responses, as evidenced by the upregulation of inflammatory cytokines [24,25].

Among gangliosides, monosialoganglioside GM1 is the best studied and most commonly used in a variety of experimental animal models to assess its therapeutic efficacy. GM1 administration exerts neuroprotective effects in experimental animal models of neurological diseases [26]. In vitro studies examining anti-inflammatory properties have shown that GM1a and GD1a gangliosides prevent TLR4 translocation to lipid rafts in LPS-stimulated PC12 cells, thus reducing the activity of TLR4 signaling pathways and inhibiting the expression of proinflammatory factors [27]. In vivo studies have revealed that the administration of GM1 to nonobese diabetic mice (NOD mice) reduces the expression of the proinflammatory factors IL-12, interferon-γ (IFN-γ), TNF-α and IL-1β, which in turn reduce inflammatory cell infiltration and diabetic symptoms [28]. In mice infected with *Trypanosoma cruzi*, GM1 reduces myocardial fibrosis and the expression of the proinflammatory factors IFN-γ and TNF-α [29]. In rats with simulated high-altitude cerebral edema (HACE), GM1 reduces the levels of proinflammatory factors IL-1β, TNF-α and IL-6 in serum and brain tissue due to hypoxia [30].

Studies have shown that exogenous GM1 gangliosides exert immunomodulatory effects; however, the efficacy of GM1 for the treatment of EIU has not been verified. In this study, we investigated the potential anti-inflammatory effects of GM1 on EIU in rats. Our in vivo and in vitro findings suggest that GM1 administration prevents ocular inflammation in rats and exerts anti-inflammatory effects on LPS-stimulated RAW 264.7 macrophages.

## 2. Materials and Methods

### 2.1. Antibodies

The following antibodies were used in this study: mouse anti-intercellular adhesion molecule-1 (anti-ICAM-1) (1:100, Cat#ab171123, Abcam, Cambridge, UK), rabbit anti-iNOS (1:250, Cat# sc-650, Santa Cruz Biotechnology, Santa Cruz, CA, USA), rabbit anti- cyclooxygenase-2 (anti-COX-2) (1:200, Cat# 15191, Abcam, Cambridge, UK), rabbit anti-TNF-α (1:200, Cat#11948, Cell Signaling, Danvers, MA, USA), mouse anti-IL-1β (1:1000, Cat# 12242, Cell Signaling, Danvers, MA, USA), rabbit anti-IL-6 (1:200, Cat#12912, Cell Signaling, Danvers, MA, USA), phospho- nuclear factor (NF)-κB p65 (1:1000, Ser536; Cat#3033, Cell Signaling, Danvers, MA, USA), rabbit anti-NF-κB p65 (1:1000, Cat#, Cell Signaling, Danvers, MA, USA), rabbit anti-p-IκB (1:1000, Ser32/36; Cat#, Cell Signaling, Danvers, MA, USA), mouse anti-IκB (1:1000, Cat#, Cell Signaling, Danvers, MA, USA), rabbit anti-p-p38 MAPK (1:1000, Thr180/Tyr182; Cat#9215, Cell Signaling, Danvers, MA, USA), rabbit anti-p38 mitogen-activated protein kinase (MAPK) (1:1000, Cat#9212, Cell Signaling, Danvers, MA, USA), rabbit anti-p- c-Jun N-terminal kinase (JNK) (1:1000, Thr183/Tyr185; Cat#4668, Cell Signaling, Danvers, MA, USA), rabbit anti-JNK (1:1000, Cat#9252, Cell Signaling, Danvers, MA, USA), rabbit anti-p-extracellular-signal-regulated kinase (ERK) (1:1000, Thr202/Tyr204; Cat#4377, Cell Signaling, Danvers, MA, USA), rabbit anti-ERK (1:1000, Cat#9102, Cell Signaling, Danvers, MA, USA), rabbit anti-β-actin (1:1000, Cat#8457, Cell Signaling, Danvers, MA, USA), fluorescein isothiocyanate (FITC)-conjugated goat anti-rabbit IgG (1:250, Cat#A11034, Invitrogen, Carlsbad, CA, USA), and horseradish peroxidase-conjugated anti-rabbit (1:10,000, Cat#111-035-003) or anti-mouse IgG (1:10,000, Cat#115-035-003, Jackson ImmunoResearch Laboratories, West Baltimore Pike, PA, USA). Biotinylated goat anti-rabbit IgG (1:250, Cat# BA-1000) and anti-mouse IgG (1:250, Cat# MKB-2225) were obtained from Vector Laboratories (Burlingame, CA, USA).

### 2.2. Animal Care

We purchased eight-week-old male Lewis rats (180–220 g) from BioLASCO Taiwan Co., Ltd. (Taipei, Taiwan). All experiments involving animals in the study were conducted in accordance with the guidelines of the Association for Research in Vision and Ophthalmology (ARVO) statement for the use of animals in ophthalmic and vision research and were approved by the Institutional Animal Care and Use Committee of the National Defense Medical Center, Taipei, Taiwan (Approval Number: IACUC-19-098; Approval date: 27 May 2019).

### 2.3. Endotoxin-Induced Uveitis (EIU) and Experimental Design

EIU was induced by subcutaneously injecting LPS (Cat# L6511, Sigma-Aldrich, St. Louis, MO, USA) into the footpad as described previously [9,31]. A dose of 200 μg of LPS was dissolved in phosphate-buffered saline (PBS, pH 7.4), and 100 μg was injected into each footpad. GM1 ganglioside (Cat# OG03918, Carbosynth Ltd., Berkshire, UK) was dissolved in PBS with 0.1% dimethyl sulfoxide (DMSO, Sigma-Aldrich, St. Louis, MO, USA) and injected intraperitoneally (i.p.) to each rat at a dose of 25 mg/kg. Rats were randomly allocated into the following four groups: (1) vehicle control group (*n* = 6)—rats received i.p. injections of vehicle (0.1% DMSO in PBS) daily for 3 days, and then the footpad was injected with 0.2 mL of PBS for 24 h; (2) GM1 group (*n* = 6)—rats received daily i.p. injections of GM1 for 3 days, and then the footpad was injected with 0.2 mL of PBS for 24 h; (3) LPS group (*n* = 6)—rats received vehicle daily for 3 days, and then the footpad was injected with LPS for 24 h; (4) GM1+LPS group (*n* = 6)—rats daily received i.p. injections of GM1 (25 mg/kg) for 3 days, and then the footpad was injected with LPS for 24 h.

### 2.4. Evaluation of Clinical Manifestations

Rats were anesthetized 24 h after the LPS or PBS injection, and inflammation in the anterior segment of the eye was photographed through a microscope equipped with a digital camera (Nikon D3100, Tokyo, Japan). The ophthalmic scoring of EIU was performed as described in previous reports [32,33] with some modifications. The severity of EIU was graded using a score ranging from 0 to 4 points as follows: Grade 0 = no obvious inflammatory response; Grade 1 = discrete inflammation of the iris and conjunctival vessels; Grade 2 = moderate dilatation of the iris and conjunctival vessels with a moderate anterior chamber flare; Grade 3 = intense iris congestion with an intense flare in the anterior chamber; Grade 4 = the same clinical signs as Grade 3 plus fibrous exudates or pupillary constriction.

### 2.5. Determination of Cell Counts and Protein Concentration in the AqH

After removing the eyeball, the cornea was punctured with a 30-gauge needle, and anterior chamber aqueous humor (AqH) was collected. A 5 μL aliquot of AqH was mixed 1:1 with a Trypan Blue solution (Sigma-Aldrich, St. Louis, MO, USA) to exclude the dead cells, and 10 μL of the cell suspension was added to a hemocytometer. The number of cells per square was calculated manually using a light microscope. The average cell counts from 5 squares for each sample was multiplied by 2 to correct for previous dilution. The remaining AqH was centrifuged at 15,000 rpm for 15 min at 4 °C, and the supernatant was collected for protein concentration analysis, which was measured using a BCA protein assay reagent kit (Thermo Fisher Scientific, Rockford, IL, USA) according to the manufacturer’s instructions.

### 2.6. Histopathological Evaluation of Rats with EIU

The eyes were immersed in Davidson’s fixative for 24 h and then embedded in paraffin. Tissue sections (4 μm thickness) were cut using a microtome designed for paraffin sections (Thermo Fisher Scientific, Rockford, IL, USA) and stained with hematoxylin and eosin (H&E). The tissue of the anterior segment of the eye was observed and scored to assess inflammatory conditions as described previously [34].

The histopathological evaluation of the inflammatory state was graded from 0 to 3. Grade 0 = no infiltrating cells; Grade 1 = mild cellular infiltration (equal to or less than 75 inflammatory cells and greater than 0); Grade 2 = moderate cellular infiltration (equal to or less than 150 inflammatory cells and greater than 75 inflammatory cells); Grade 3 = severe cellular infiltration and severe anterior chamber exudates (more than 150 inflammatory cells).

### 2.7. Immunohistochemistry

Whole eyes were fixed with Davidson’s fixative for 24 h and then subjected to routine paraffin sectioning. Paraffin sections were deparaffinized with xylene and subjected to antigen retrieval with EnVision FLEX Target Retrieval Solution (low pH) (K8005, Dako, Glostrup, Denmark) at 95 °C for 15 min. Endogenous peroxidase activity was blocked by treating sections with 3% hydrogen peroxide for 20 min. After washes with TBS, nonspecific binding was blocked by incubating the sections with blocking solution (3% bovine serum albumin and 0.3% Triton X-100 in TBS) for 30 min. The sections were incubated with the primary antibody overnight at 4 °C. After washing with TBS, the sections were incubated with a secondary antibody (goat anti-rabbit or mouse IgG conjugated with horseradish peroxidase) for 1 h. Staining was visualized using 3,3′-diaminobenzidine (DAB, Sigma-Aldrich, St. Louis, MO, USA) as the chromogen and hematoxylin (Muto Pure Chemicals Co., Ltd., Tokyo, Japan) for counterstaining.

### 2.8. RAW 264.7 Cell Culture

The RAW 264.7 cell line (third passage at the time of purchase) was purchased from American Type Culture Collection (ATCC, Manassas, VA, USA) and maintained in growth medium composed of Dulbecco’s modified Eagle’s medium (DMEM) supplemented with 10% fetal bovine serum (FBS), 100 μg/mL streptomycin, and 100 U/mL penicillin in 100 mm Petri dishes (BD Falcon, Franklin Lakes, NJ, USA) in a humidified atmosphere containing 5% CO_2_ at 37 °C. The medium was changed every 2 days, and the cells were never cultured beyond passage 20. RAW 264.7 cells (2.6 × 10^4^ cells/cm^2^) were seeded onto glass coverslips or into six-well plates. The stock solution of GM1 was dissolved in DMSO. The final concentration of DMSO did not exceed 0.1% in the culture medium. The cultures were pretreated with GM1 for 1 h before the addition of LPS (1 μg/mL).

### 2.9. Cell Viability Assays

RAW 264.7 cell viability (2.6 × 10^4^ cells/cm^2^) was determined using a cell counting kit (CCK-8) assay (Dojindo, Kumamoto, Japan). Briefly, CCK-8 solution (1:40) was added to each well of a 24-well plate; the cells were then incubated at 37 °C for another 2 h, and absorbance was measured at 450 nm using a microplate reader.

### 2.10. Enzyme-Linked Immunosorbent Assay (ELISA)

Culture supernatant was harvested at the indicated time and mouse TNF-α, IL-1β and IL-6 were detected with enzyme-linked immunosorbent assay (ELISA) kits (Bioss Antibodies Inc., Woburn, MA, USA) according to the manufacturer’s instructions. The optical density was monitored at 450 nm using a microplate reader.

### 2.11. RT-qPCR Analysis

Total RNA from cell cultures was extracted with Trizol reagent (Invitrogen, Carlsbad, CA, USA). cDNA was synthesized by a High-Capacity cDNA Reverse Transcription Kit (Applied Biosystems, Waltham, MA, USA). Quantitative real-time PCR (qPCR) was carried out with TaqMan Gene Expression Master Mix (Applied Biosystems, Waltham, MA, USA) according to the manufacturer’s instructions. β-actin served as the housekeeping gene. Primer-probe sets were as follows: β-actin (Mm00607939_s1); TNF-α (Mm00443258_m1); IL-1β (Mm00434228_m1); and IL-6 (Mm00446190_m1); website link: https://www.thermofisher.com/tw/zt/home.html (accessed on 22 February 2022).

### 2.12. Western Blot

Cells or tissues were homogenized in radioimmunoprecipitation assay (RIPA) buffer containing 1 mM phenylmethanesulfonyl fluoride (PMSF), 5 mM NaF and a protease inhibitor cocktail. The supernatant was collected after centrifugation at 12,000 rpm for 10 min at 4 °C, and the protein concentration was determined with a bicinchoninic acid (BCA) kit. A total of 35 μg of the protein extract was loaded onto 10–15% SDS–PAGE gels. The proteins were transferred to polyvinylidene difluoride (PVDF) membranes after electrophoresis. The membranes were blocked with a blocking solution (TBS-T, 5% nonfat milk in TBS with 0.1% Tween 20) for 1 h at room temperature, incubated with primary antibodies at 4 °C overnight, and then incubated with horseradish peroxidase (HRP)-conjugated secondary antibodies (1:10,000) for 1 h. The membranes were washed with TBS-T then incubated with enhanced chemiluminescence reagents (ECL, Thermo Fisher Scientific, Rockford, IL, USA) to visualize the bands. Each experiment was repeated at least three times. Immunoreactive bands were subjected to densitometry analysis. Images of each blot were quantified using ImageJ software (Version 1.53f, NIH, Bethesda, MD, USA). β-actin (1:2000) was used to normalize protein loading.

### 2.13. Immunofluorescence Staining

RAW 264.7 cells (2.6 × 10^4^ cells/cm^2^) growing on coverslips were fixed with 10% neutral buffered formalin for 10 min. Cells were permeabilized and blocked with blocking buffer (0.5% nonfat milk and 0.2% Triton X-100 in PBS) for 15 min, followed by incubation with the primary antibodies overnight at 4 °C. The cells were rinsed with PBS and then incubated with the secondary antibody (FITC-conjugated rabbit or mouse anti-goat IgG) at room temperature for 1 h. The nuclei were labeled with 4′,6-diamidino-2-phenylindole (DAPI, 1 μg/mL; Catalog number 40043, Biotium Inc., Fremont, CA, USA) for 10 min. Then, the coverslips were mounted on slides with mounting medium (3% n-propyl gallate and 50% glycerol in PBS) and observed under a fluorescence microscope (Nikon, Tokyo, Japan).

### 2.14. Detection of Reactive Oxygen Species (ROS)

Total ROS level was determined by 2′,7′-dichlorodihydrofluorescein diacetate (DCFH-DA) staining. Nicotinamide adenine dinucleotide phosphate hydrogen (NADPH) oxidase inhibitor diphenyleneiodonium (DPI) was obtained from Cayman Chemical Company (Ann Arbor, MI, USA). At the end of the treatment, cells were loaded with 10 μM DCFH-DA (Sigma-Aldrich, St. Louis, MO, USA) and incubated at 37 °C for 20 min. Cells were washed with PBS and images were captured under a fluorescence microscope with excitation/emission 485/535 nm. The captured images were analyzed by ImageJ software to measure fluorescence intensity. For each image, the corrected total cell fluorescence (CTCF) was calculated using the formula [35]:

CTCF = Average Integrated intensity − (Average selected area × Average mean of background).(1)

Mean fluorescence of background was recorded from randomly selected square areas outside of the area of interest and at least three independent experiments were run for each condition.

### 2.15. Statistics

Quantitative data are presented as the mean ± standard deviation (SD) for at least three independent experiments. One-way analysis of variance (ANOVA) was used to analyze the data and the statistical significance was determined by Bonferroni’s post hoc test. Differences were considered significant at * *p* < 0.05 and highly significant at ** *p* < 0.01.

## 3. Results

### 3.1. Effect of GM1 on the Inflammatory Manifestation of EIU in Rats

**Figure 1:**
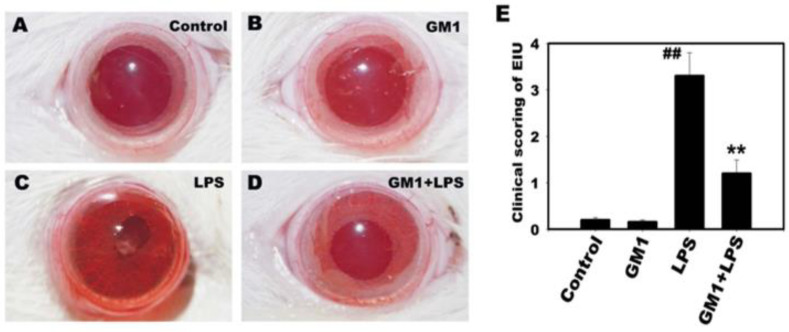
Effect of GM1 on the inflammatory manifestation of EIU in rats. Lewis rats were pretreated with GM1 and then treated with or without LPS for another 24 h. Representative ocular images of the anterior segment for four different treatment groups: control (**A**), GM1 alone (**B**), LPS (**C**), and GM1+LPS (**D**). (**E**) The inflammatory response was significantly reduced in the GM1 pretreatment group compared to the LPS group in terms of clinical scores. Data are presented as the mean ± SD. ^##^ *p* < 0.01 compared with the control group; ** *p* < 0.01 compared with the LPS treatment group. Each group contained *n* = 6 rats.

First, we examined the effects of GM1 on the clinical manifestations of EIU in rats. The rats were pretreated with or without GM1 prior to the LPS injection. After 24 h, inflammatory symptoms in the anterior segment of the eyes were observed and imaged. As shown in Figure 1, no inflammatory response was observed in the control group (Figure 1A) or the GM1 alone group (Figure 1B). In the LPS group, conjunctival edema, ciliary congestion and iris vasodilatation (erythema or redness) were observed in rats 4 to 6 h after the LPS injection. At 12 to 16 h, fibrous exudates and occlusion of the pupil were observed, which peaked at 22–24 h (Figure 1C). However, these clinical symptoms were alleviated in the GM1+LPS group (Figure 1D). The pathological severity was assessed as a score ranging from 0 to 4 according to the degree of inflammation; a higher score indicates a greater severity. GM1 significantly attenuated LPS-induced clinical EIU scores compared to those of the LPS-treated group (Figure 1E).

### 3.2. Effects of GM1 on LPS-Induced Cellular Infiltration and Protein Concentration in the AqH of Rats with EIU

**Figure 2:**
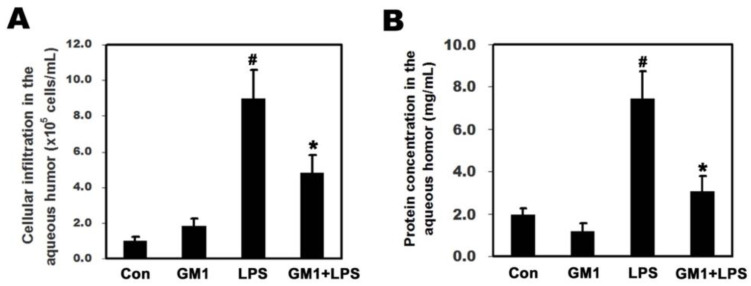
Effects of GM1 on cellular infiltration and protein concentration in the AqH of rats with EIU. Rats were pretreated with GM1 and then treated with or without LPS for another 24 h. The AqH was collected from each group, cells were counted, and the protein concentration was measured by a blinded observer. GM1 significantly decreased cellular infiltration (**A**) and protein concentration (**B**) in the AqH of EIU rats. The data are presented as the mean ± SD. ^#^ *p* < 0.05 compared with the control group; * *p* < 0.05 compared with the LPS-treated group. *n* = 6 rats per group. AqH: aqueous humor.

The pathological characteristics of EIU include ocular infiltration by inflammatory cells and leakage of protein into the AqH of the eye. We collected AqH to count cells and measure the protein concentration to investigate the anti-inflammatory effect of GM1. As shown in Figure 2A, the number of infiltrating cells in the AqH was significantly increased in the LPS group compared to that in the control group. However, in the GM1+LPS group, the number of infiltrating cells was decreased significantly compared to the LPS group. Similarly, the protein concentration in the AqH of the LPS group increased significantly compared to that in the control group; however, in the GM1+LPS group, this increase in protein concentration was significantly attenuated (Figure 2B) compared to that in the LPS group. Based on these results, GM1 attenuates the LPS-induced decrease in the integrity of the blood-ocular barrier in rats with EIU.

### 3.3. Effects of GM1 on the Histopathological Changes Adjacent to the Iris-Ciliary Body (ICB) of EIU Rats

**Figure 3:**
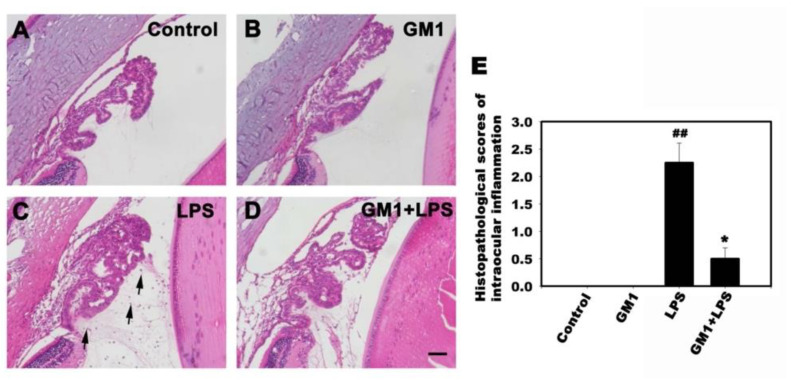
Effects of GM1 on the histological changes in the ICB of rats with EIU. Rats were pretreated with GM1 and then treated with or without LPS for another 24 h. Representative images of H&E staining of the ICB from the four different treatment groups: vehicle control (**A**), GM1 alone (**B**), LPS (**C**) and GM1+LPS (**D**). The LPS-treated rats showed increased leukocyte infiltration in the ICB and the AqH (arrows in C), whereas the number of infiltrating cells was reduced by GM1. (**E**) The severity of histological changes was evaluated by histopathological scoring with a scale ranging from 0 to 3. GM1 significantly attenuated LPS-induced pathological scores in the ICB. ^##^ *p* < 0.01 compared with the control group; * *p* < 0.05 compared with the LPS-treated group. Bar = 50 μm. ICB: iris-ciliary body. AqH: aqueous humor.

The histopathological analysis of the effect of GM1 on the anterior segment of rats with EIU was performed using H&E staining. As shown in Figure 3, the morphological data indicated no cellular infiltration adjacent to the ICB of the anterior segment in either the control (Figure 3A) or GM1 alone group (Figure 3B). Conversely, in the LPS group, the histological evaluation revealed the accumulation of cells infiltrating into the anterior segment (Figure 3C). Compared with the LPS group, the GM1+LPS group showed a reduction in cellular infiltration (Figure 3D). The severity of histological changes was evaluated by histopathological scoring with a scale ranging from 0 to 3 (see the Section 2). GM1 significantly attenuated LPS-induced histopathological scores in rats with EIU (Figure 3E). Therefore, GM1 prevented LPS-induced histopathological changes in rats with EIU.

### 3.4. Effect of GM1 on the Recruitment of COX-2-Positive Cells Adjacent to the ICB in Rats with EIU

**Figure 4:**
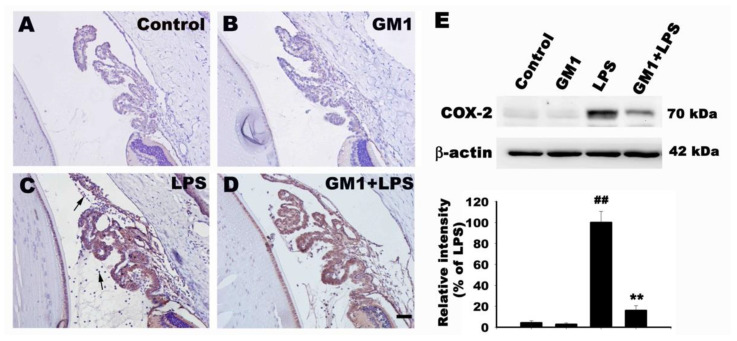
Effect of GM1 on COX-2 expression in the anterior segment of rats with EIU. Rats were pretreated with GM1 and then treated with or without LPS for another 24 h. Representative images of COX-2 immunohistochemical staining in sections from the four different treatment groups: vehicle control (**A**), GM1 alone (**B**), LPS (**C**) and GM1+LPS (**D**) at 24 h after the LPS injection. Note that abundant COX-2-positive inflammatory cells infiltrated the ICB of rats with EIU (arrows in C), and this inflammatory response was attenuated by GM1. (**E**) Western blot analysis of COX-2 levels. The protein bands from each treatment group were quantified by densitometry. GM1 significantly reduced the LPS-induced increase in COX-2 expression in the anterior segment of rats. Data are presented as percentages of the LPS-treated group (mean ± S.D., *n* = 3). ^##^ *p* < 0.01 compared with the control group; ** *p* < 0.01 compared with the LPS-treated group. Bar = 50 μm.

COX-2 is an inducible isoform of COX that is primarily expressed in inflammatory cells. COX-2 is a key enzyme required for the generation of prostaglandins from arachidonic acid. In this study, the effects of GM1 on infiltrating COX-2-positive cells were investigated in the ICB of rats with EIU using immunohistochemistry. As shown in Figure 4, few COX-2-positive cells were observed in the anterior segment of both the control (Figure 4A) and GM1 alone groups (Figure 4B); however, in the LPS group, the anterior segment exhibited a large number of infiltrating COX-2-positive cells (Figure 4C). Compared to the LPS group, the number of COX-2-positive cells was markedly decreased in the GM1+LPS group (Figure 4D). Western blot analysis further confirmed that GM1 attenuated LPS-induced COX-2 expression in the anterior segment of rats (Figure 4E). Based on these results, GM1 decreased the recruitment of COX-2-positive cells adjacent to the ICB in rats with EIU.

### 3.5. Effects of GM1 on ICAM-1 Expression in the ICB of Rats with EIU

**Figure 5:**
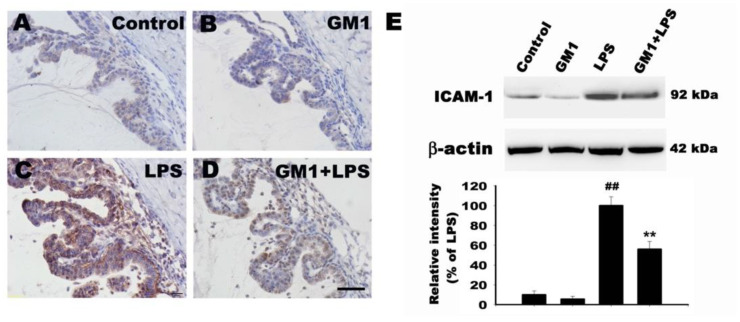
Effect of GM1 on ICAM-1 expression in the ICB of rats with EIU. Rats were pretreated with GM1 and then treated with or without LPS for another 24 h. Representative images of ICAM-1 immunohistochemical staining in sections from the four different treatment groups at 24 h after the LPS injection: vehicle control (**A**), GM1 alone (**B**), LPS (**C**) and GM1+LPS (**D**). (**E**) Western blot analysis of ICAM-1 levels. The protein bands from each treatment group were quantified by densitometry. GM1 significantly reduced LPS-induced increases in ICAM-1 expression. Data are presented as percentages of the LPS-treated group (mean ± S.D., *n* = 3). ^##^ *p* < 0.01 compared with the control group; ** *p* < 0.01 compared with the LPS-treated group. Bar = 50 μm. ICB: iris-ciliary body.

Previous studies have reported an important role for increased ICAM-1 expression in inflammatory cell infiltration during EIU [36]. We examined the effects of GM1 on ICAM-1 expression in the ICB of rats with EIU using immunohistochemical staining. As shown in Figure 5, the ICB constitutively expressed low levels of ICAM-1 in both the control (Figure 5A) and GM1 alone groups (Figure 5B); however, in the LPS group, the ICB expressed high levels of ICAM-1 (Figure 5C). Compared to the LPS group, ICAM-1 expression was markedly reduced in the GM1+LPS group (Figure 5D). Western blot analysis further confirmed that GM1 attenuated LPS-induced ICAM-1 expression in rats (Figure 5E). The results indicated that GM1 reduced ICAM-1 expression and contributed to attenuating the recruitment of inflammatory cells adjacent to the ICB in rats with EIU.

### 3.6. Effect of GM1 on the Transcribed and Secreted Level of Proinflammatory Factors in LPS-Stimulated RAW 264.7 Cells

**Figure 6:**
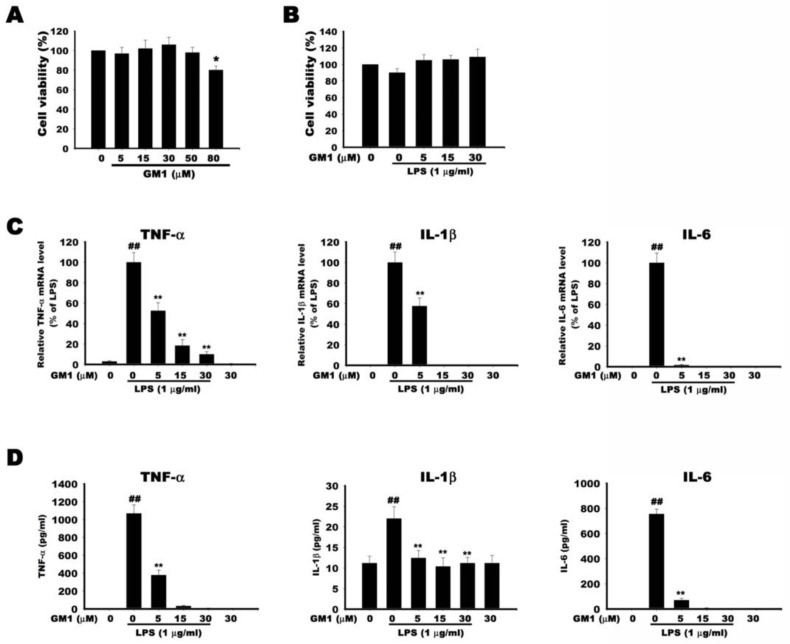
Effect of GM1 on the transcribed and secreted levels of proinflammatory mediators in LPS-stimulated RAW 264.7 cells. (**A**,**B**) Effect of GM1 with or without LPS stimulation on cell viability. Cells were treated alone with different concentrations of GM1 as indicated (**A**) or pretreated with GM1 for 1 h and then stimulated with LPS (1 μg/mL) for 24 h (**B**). Cell viability was determined using a CCK-8 assay. Quantitative data are presented as percentages of the corresponding untreated control value (mean ± S.D., *n* = 3, quadruplicate wells for each condition). (**C**) Cells were pretreated with different concentrations of GM1 (5, 15, 30 µM) and then stimulated with LPS for 6 h. mRNA expression levels for TNF-α, IL-1β and IL-6 were measured by RT-qPCR. (**D**) Cells were pretreated with different concentration of GM1 (5, 15, 30 µM) and then stimulated with LPS for 24 h. Secreted levels of TNF-α, IL-1β and IL-6 were determined by ELISA. ^##^ *p* < 0.01 compared with the control group; * *p* < 0.05 compared with the LPS-treated group; ** *p* < 0.01 compared with the LPS-treated group.

First, we examined the cytotoxicity of GM1. RAW 264.7 cells were treated with GM1 at concentrations ranging from 0 μM to 80 μM for 24 h, and cell viability was measured using the CCK-8 assay. As shown in Figure 6A, GM1 (up to 50 µM) did not affect cell viability. Therefore, subsequent experiments were conducted with GM1 at concentrations less than 30 µM. Moreover, GM1 (5, 15 and 30 µM) does not cause cytotoxicity to cells in the presence of LPS (1 µg/mL) (Figure 6B).

TNF-α, IL-1β and IL-6 are the main proinflammatory mediators in response to LPS stimulation. We investigated the effect of GM1 (5, 15 and 30 µM) on these factors in LPS-stimulated RAW 264.7 cells. The transcribed and secreted levels of these factors were determined by RT-qPCR and ELISA, respectively. As shown in Figure 6, mRNA (Figure 6C) and secreted levels (Figure 6D) of TNF-α, IL-1β and IL-6 were markedly increased upon exposure to LPS. However, GM1 significantly suppressed the effects in a dose-dependent manner, and this anti-inflammatory effect was not due to cytotoxicity.

### 3.7. Effect of GM1 on the Protein Level of Proinflammatory Enzymes and Factors in LPS-Stimulated RAW 264.7 Cells

**Figure 7:**
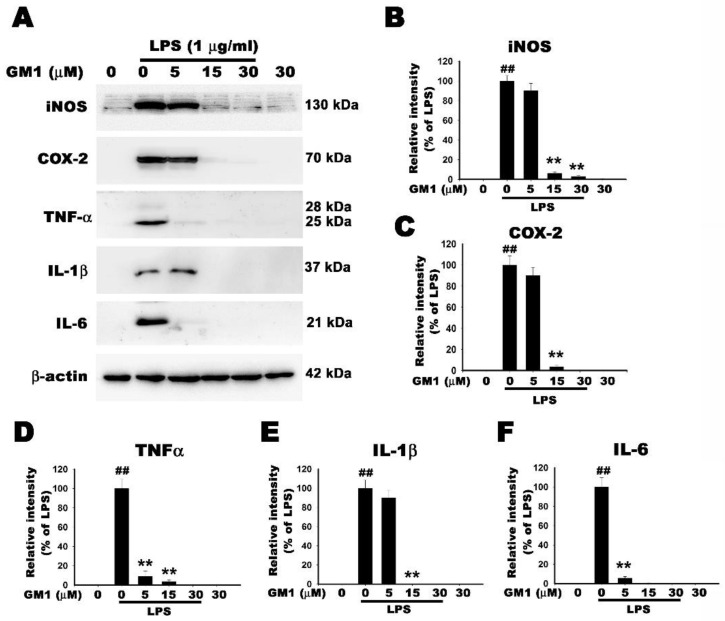
Effect of GM1 on the protein levels of proinflammatory mediators in LPS-stimulated RAW 264.7 cells. (**A**) Cells were pretreated with different concentrations of GM1 (5, 15, 30 μM) for 1 h, stimulated with LPS (1 μg/mL) for 8 h, and then subjected to Western blot analysis with anti-iNOS, anti-COX-2, anti-TNF-α, anti-IL-1β and IL-6 antibodies; β-actin was used as an internal control. (**B**–**F**) The protein bands from each treatment group were quantified using densitometry. Data are presented as percentages of the LPS-treated group (mean ± S.D., *n* = 3). ^##^ *p* < 0.01 compared with the control group; ** *p* < 0.01 compared with the LPS-treated group.

The main proinflammatory enzymes involved in the synthesis of NO and PGE2 are iNOS and COX-2, respectively. Therefore, we examined the effect of GM1 on the expression of the iNOS and COX-2 proteins in LPS-stimulated RAW 264.7 cells using Western blot analysis. As shown in Figure 7A–C, the expression of iNOS and COX-2 was induced in LPS-stimulated RAW 264.7 cells. GM1 pretreatment significantly inhibited the LPS-induced increases in expression in a dose-dependent manner (0–30 μM) compared to the control group, whereas treatment with GM1 alone had no effects on the expression of these proteins (Figure 7A–C). The effect of GM1 on LPS-induced production of proinflammatory mediators was further examined using Western blot. As shown in Figure 7A, LPS administration markedly induced the production of proinflammatory cytokines, including TNF-α, IL-1β and IL-6; in contrast, pretreatment with GM1 decreased the production of these cytokines in a dose-dependent manner (Figure 7D–F). Treatment with GM1 alone (30 μM) had no effect on the production of these proinflammatory cytokines compared to the control group (Figure 7D–F). Thus, GM1 exerts an anti-inflammatory effect on LPS-stimulated RAW 264.7 cells.

### 3.8. Effect of GM1 on NF-κB Activation in LPS-Stimulated RAW 264.7 Cells

**Figure 8:**
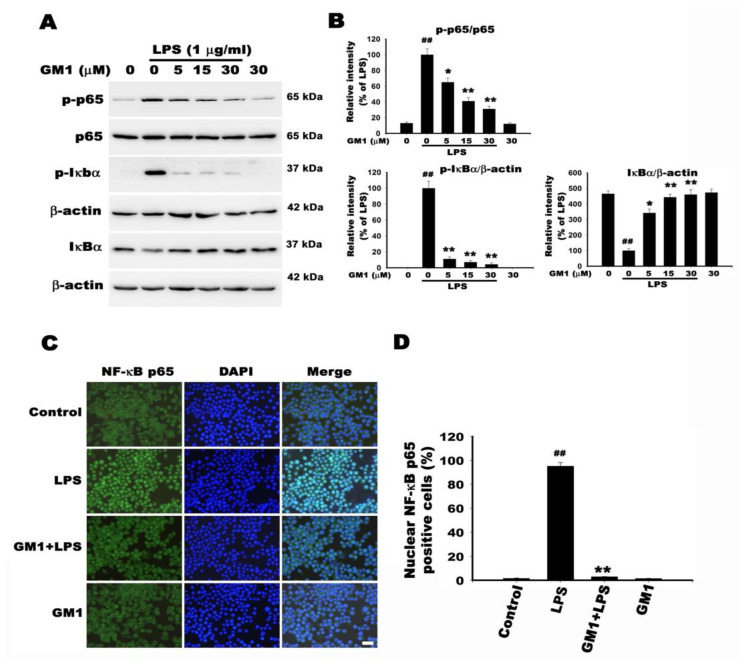
Effect of GM1 on NF-κB activation in LPS-stimulated RAW 264.7 cells. (**A**) Cells were pretreated with different concentrations of GM1 (5, 15, 30 μM) for 1 h and then stimulated with LPS (1 μg/mL) for 1 h. Western blot analysis was performed to determine the levels of the p-p65, p65, p-IκB and IκB proteins. β-Actin was used as an internal control. (**B**) Quantitative analysis of protein levels. Data are presented as percentages relative to the LPS group (mean ± SD, *n* = 3). ^##^ *p* < 0.01 compared with the control group; * *p* < 0.05 and ** *p* < 0.01 compared with the LPS treatment group. (**C**) Immunofluorescence analysis of NF-κB nuclear translocation. Cells were pretreated with GM1 (30 μM) for 1 h and then administered LPS (1 μg/mL) for 1 h. Nuclear translocation of NF-κB p65 was determined by performing immunofluorescence staining using anti-p65 NF-κB antibodies. Nuclei (blue) were stained with DAPI. Bar = 30 μm. (**D**) Percentage of cells with nuclear NF-κB p65. Quantitation of NF-κB p65 nuclear translocation in the indicated groups. Data are reported as percentages of p65 nuclear-positive cells among 100 cells per sample (mean ± S.D., *n* = 3). ^##^ *p* < 0.01 compared with the control group; * *p* < 0.05 compared with the LPS treatment group; ** *p* < 0.01 compared with the LPS treatment group.

NF-κB activation and nuclear translocation play a key role in the production of iNOS, COX-2 and proinflammatory factors [37]. We investigated the effect of GM1 on NF-κB activation in LPS-stimulated RAW 264.7 cells. NF-κB activation was determined by measuring the phosphorylation of p65 and IκB and degradation of IκB. As shown in Figure 8A,B, the phosphorylation of p65 was significantly increased in LPS-stimulated RAW 264.7 cells. GM1 pretreatment dose-dependently inhibited the LPS-induced increase in p-p65 levels. In addition, LPS administration promoted IκB degradation via IκB phosphorylation, whereas GM1 attenuated LPS-induced IκB phosphorylation in a dose-dependent manner while attenuating IκB degradation. Treatment with GM1 alone (30 µM) had no effect on NF-κB activation (Figure 8A,B). Immunofluorescence staining (Figure 8C) further confirmed the inhibitory effect of GM1 on the translocation of NF-κB p65 to the nucleus. In the control group of cells, NF-κB p65 was mainly located in the cytoplasm. In contrast, a significant increase in nuclear NF-κB p65 immunofluorescence staining was observed after 1 h of LPS stimulation. After pretreatment with 30 µM GM1, NF-κB p65 translocation to the nucleus was inhibited, whereas GM1 administration alone did not affect its cytoplasmic and nuclear distribution (Figure 8C,D). Therefore, GM1 reduces the production of proinflammatory enzymes and proinflammatory cytokines by inhibiting NF-κB activation and nuclear translocation.

### 3.9. Effect of GM1 on MAPK Activation in LPS-Stimulated RAW 264.7 Cells

MAPKs, including p38, JNK and ERK, are associated with the production of proinflammatory factors and NF-κB activation [38,39]. Therefore, we analyzed the effect of GM1 on LPS-induced MAPK activation by using Western blotting to detect the phosphorylation of p38, JNK, and ERK.

**Figure 9:**
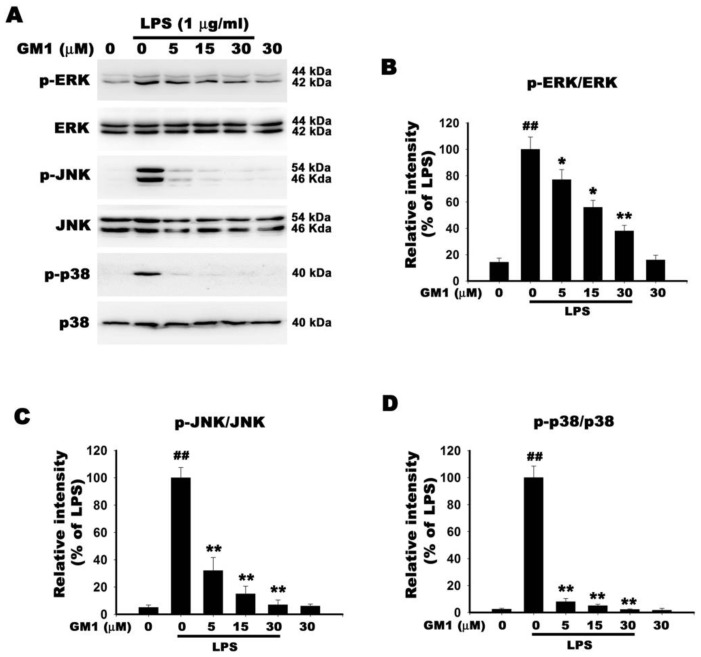
Effect of GM1 on MAPK activation in LPS-stimulated RAW 264.7 cells. (**A**) Cells were pretreated with various concentrations of GM1 (5, 15, 30 μM) for 1 h and then stimulated with LPS (1 μg/mL) for 1 h. Cell lysates were subjected to Western blotting to determine levels of MAPK proteins (p-ERK/ERK, p-JNK/JNK and p-p38/p38). (**B**–**D**) Quantitative analysis of MAPK protein levels. Data are presented as percentages relative to the LPS group (mean ± SD, *n* = 3). ^##^ *p* < 0.01 compared with the control group; * *p* < 0.05 and ** *p* < 0.01 compared with the LPS treatment group.

As shown in Figure 9, LPS stimulation of RAW 264.7 cells resulted in the phosphorylation of ERK, JNK and p38. Concentration-dependent reductions in LPS-induced ERK, JNK and p38 phosphorylation were observed after treatment with GM1 (0–30 μM). GM1 alone (30 μM) had no effect on ERK, JNK and p38 phosphorylation. The results indicated that GM1 pretreatment inhibited LPS-induced activation of MAPK signaling pathways.

### 3.10. Effect of GM1 on ROS Production by LPS-Stimulated RAW 264.7 Cells

**Figure 10:**
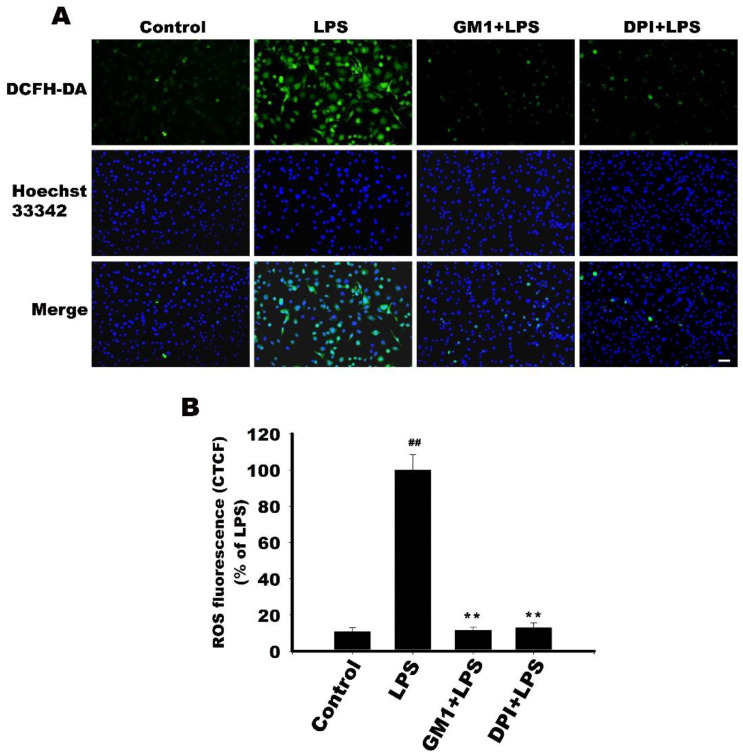
Effect of GM1 on LPS-stimulated ROS production by LPS-stimulated RAW 264.7 cells. (**A**) Cells were pretreated with 30 μM GM1 or 500 nM DPI and then stimulated with LPS for 9 h. Intracellular ROS level was measured using the DCFH-DA assay. Bar = 50 μm. (**B**) Quantification of ROS level. Staining intensity was measured as corrected total cell fluorescence (CTCF). Data are expressed as a percentage of the LPS-treated group (mean ± SD, *n* = 3). ^##^ *p* < 0.01 vs. control group; ** *p* < 0.01 vs. LPS-treated group.

Macrophages, the first line of defense, are responsible for the production of reactive oxygen species (ROS) at the onset of exposure to inflammatory stimuli [40]. In LPS-treated RAW 264.7 cells, a moderate level of ROS can act as a second messenger to initiate an inflammatory response [41]. LPS stimulates ROS production through membrane-bound NADPH oxidase (NOX) activation [42,43,44]. NOX-dependent ROS production was associated with the activation of NF-κB and MAPK signaling, which is required for LPS-induced macrophage activation [40,43,45]. In the present study, we determined the effect of GM1 on ROS formation by LPS-stimulated RAW 264.7 cells using the dichlorodihydrofluorescein diacetate (DCFH-DA) assay. As shown in Figure 10, intracellular ROS levels were significantly increased after LPS treatment, whereas GM1 treatment abolished LPS-induced ROS production.

**Figure 11:**
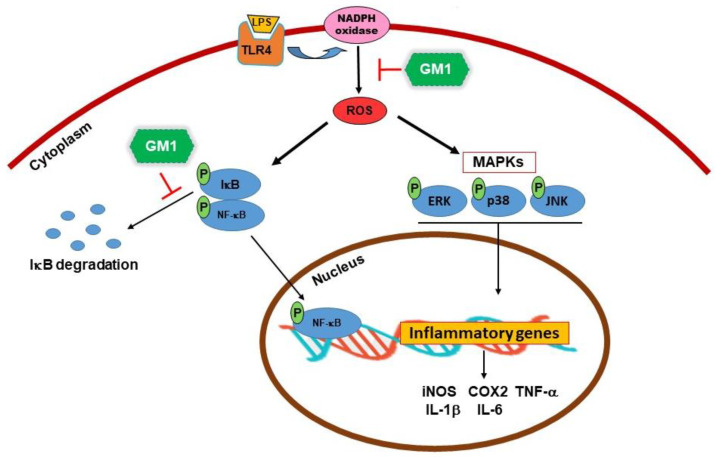
Schematic of the anti-inflammatory mechanism of GM1 ganglioside. GM1 inhibited the proinflammatory responses in LPS-treated RAW 264.7 cells by suppressing NADPH oxidase-mediated ROS production and subsequently inhibiting NF-κB and MAPK signaling pathways.

In addition, the NOX inhibitor diphenylene iodonium (DPI) also significantly inhibited LPS-induced ROS production, suggesting that NOX activation is required for LPS-induced ROS formation. GM1 inhibited the activation of NF-κB and MAPKs signaling, which may be partially attributed to its potent downregulation of NOX-mediated ROS production. Figure 11 summarizes the possible mechanisms by which GM1 inhibits inflammatory responses.

## 4. Discussion

In this study, we describe the role of exogenous GM1 ganglioside in anti-inflammatory responses. In the EIU animal model, GM1 alleviated LPS-induced clinical symptoms of uveitis, and decreased inflammatory cell infiltration and protein concentration in the AqH, as determined by biochemical examinations. Histopathological and Western blot analyses showed that GM1 attenuated LPS-induced infiltration of the anterior segment by COX-2-positive inflammatory cells that was accompanied by the downregulation of ICAM-1, a critical adhesion molecule expressed in the ciliary body that is responsible for the recruitment of inflammatory cells. Experiments aimed at elucidating the anti-inflammatory mechanism showed that GM1 completely inhibited LPS-induced production of proinflammatory mediators by RAW 264.7 macrophages by suppressing the activation of NF-κB and MAPKs, suggesting that GM1 is a promising candidate drug for inflammation-mediated ocular diseases.

The pathology of uveitis is basically caused by excessive inflammatory conditions [46]. LPS is one of the most effective stimulants that triggers proinflammatory responses. EIU is a well-established model for investigating the pathological mechanism of ocular inflammation and evaluating the therapeutic efficacy of potential anti-inflammatory agents. Administration of LPS induces the infiltration of inflammatory cells in the ICB and increases protein concentration in the AqH of rats with EIU, accompanied by clinical pathological symptoms, including conjunctival chemosis (edema), erythema (redness) and ocular discharge. However, GM1 alleviated these clinical symptoms and reduced LPS-induced increases in protein and the number of infiltrating cells, indicating the anti-inflammatory effects of GM1.

Recruitment and activation of inflammatory cells in the ICB is the primary pathogenesis of EIU. These inflammatory cells mainly include neutrophils and macrophages. Immune cells release proinflammatory mediators that modulate the pathological progression of uveitis. Among these mediators, prostaglandin is a critical proinflammatory mediator. Prostaglandin is produced by COX-2; therefore, COX-2-positive cells are useful as an indicator of the presence of inflammatory cells. In this study, abundant COX-2-positive cells infiltrated into the anterior chamber and ICB tissue of rats with EIU, whereas GM1 reduced the recruitment of these cells and attenuated the expression of the COX-2 protein. A reduction in the infiltration of inflammatory cells may be one of the significant effects of GM1 for alleviating uveitis.

The expression of ICAM-1, an adhesion protein expressed in the epithelium of the ICB, is induced by LPS [47]. ICAM-1 participates in cellular infiltration during inflammation. ICAM-1 is recognized by inflammatory cells and is involved in rolling and adhesion [36,48]. Systemic administration of anti-ICAM-1 monoclonal antibodies has been shown to inhibit LPS-induced cellular infiltration into the anterior segment of the eye [36]. In this study, GM1 reduced macrophage activation; therefore, it decreased LPS-induced inflammation and ICAM-1 expression, contributing to a reduction in the infiltration of COX-2-positive cells.

Macrophage activation is associated with the pathogenesis of several inflammatory diseases, and inhibition of macrophage activation and production of proinflammatory factors may contribute to the development of effective treatments for inflammatory diseases [49]. In LPS-stimulated RAW 264.7 cells, iNOS and COX-2 produce the proinflammatory factors NO and PGE2, respectively. The proinflammatory factors TNF-α, IL-1β and IL-6 are also associated with the development of uveitis. In the current study, GM1 significantly inhibited the production of iNOS, COX-2, TNF-α, IL-6 and IL-1β by LPS-stimulated RAW 264.7 cells. Based on these results, GM1 exerts anti-inflammatory effects in vitro.

This study investigated whether GM1 affects the activation of NF-κB to further elucidate the anti-inflammatory mechanism of GM1. NF-κB is a protein complex consisting mainly of p50 and p65 heterodimers that is an inducible transcription factor regulating DNA transcription and proinflammatory factor production during inflammation. In the inactivated state, NF-κB activity is inhibited by a physical interaction with IκBα, whereas upon stimulation by LPS, IκBα is phosphorylated by IκB kinase and dissociates from NF-κB, and is subsequently degraded via the ubiquitin–proteasome pathway. Free p50 and p65 are activated, and phosphorylated p65 and p50 are then translocated to the nucleus where they function as transcription factors to induce the expression of proinflammatory genes [50,51]. In the present study, GM1 inhibited the LPS-induced phosphorylation and degradation of IκBα in RAW 264.7 cells along with phosphorylation of NF-κB p65 and nuclear translocation. Therefore, GM1 can suppress LPS-induced expression of proinflammatory molecules by inhibiting the activation of NF-κB p65.

MAPKs are a family of serine/threonine protein kinases that modulate various cellular processes in response to external stress signals [52]. Downstream activation of MAPKs is associated with the production of proinflammatory factors; therefore, they are considered potential therapeutic targets for anti-inflammation [53,54]. Activated MAPKs phosphorylate various substrate proteins, including proinflammatory transcription factors such as c-Jun, c-Fos, Elk-1, activated transcription factor 2 (ATF2) and cardiomyocyte enhancer factor, and positively regulate gene transcription [55]. In macrophages, LPS-stimulated TLR4 signaling increases the phosphorylation of MAPKs, including JNK, p38 MAPK and ERK, which increases the expression of proinflammatory and chemotactic factors and promotes inflammatory responses [56]. In addition, MAPKs participate in the positive regulation of NF-κB transcriptional activity [57]. In the present study, GM1 pretreatment attenuated the LPS-induced phosphorylation of JNK, p38 MAPK and ERK in RAW 264.7 cells. The results suggest that inhibition of MAPK activity by GM1 in LPS-stimulated RAW 264.7 cells is associated with its anti-inflammatory activity.

ROS act as signaling molecules triggering various biological responses upon exposure to stressful environments [40,58]. At moderate (non-toxic) levels of ROS, they can be considered as a second messenger necessary for the initiation of the inflammatory response and to maintain cellular homeostasis. However, excessive ROS production is thought to be critical for the production of various inflammatory mediators and the progression of tissue damage [40]. Therefore, modulating ROS production and oxidative stress would be an effective strategy for treating inflammatory diseases. In macrophages, LPS can trigger ROS production via NOX activation at the cell membrane [41,43,44]. Inhibition of NOX with DPI can prevent ROS production and activation of NF-κB and MAPKs, thus attenuating the inflammatory response [41,43,45]. In the present study, LPS-induced ROS production by RAW 264.7 cells was significantly inhibited by GM1. It is likely that GM1 may inhibit LPS-induced activation of NF-κB and MAPKs in part by reducing NOX-dependent ROS production. Previous studies have indicated the anti-inflammatory activity of several gangliosides (e.g., GM3 and GD1a) in LPS-stimulated RAW 264.7 macrophages [59,60]. In the present study, GM1 exerted an anti-inflammatory effect on LPS-stimulated RAW 264.7 cells. Nonsteroidal anti-inflammatory drugs (naproxen, ibuprofen and aspirin) are commonly used for anti-inflammation, but their prolonged use may cause gastric ulcers, kidney damage, stroke or heart attack [61]. Corticosteroids are still the standard treatment for uveitis. However, corticosteroids may cause many adverse ocular side effects, including accelerated cataracts and increased intraocular pressure [62], as well as systemic side effects, such as Cushing’s syndrome, hypertension, hyperglycemia and osteoporosis [14,63]. Recently, GM1 was found to ameliorate chemotherapy-induced peripheral neurotoxicity in a phase III clinical trial [64], suggesting its safety and applicability, but the detailed anti-inflammatory mechanism of GM1 requires further investigation.

## 5. Conclusions

The present study revealed a potential inhibitory effect of GM1 on the inflammatory symptoms in the eye of an animal model of EIU. In addition, GM1 inhibited the production of proinflammatory factors by LPS-stimulated RAW 264.7 macrophages by suppressing the NADPH oxidase-mediated ROS production, and subsequently inhibiting NF-κB and MAPK signaling pathways. These observations suggest that GM1 gangliosides have the potential to inhibit macrophage activation and infiltration by preventing ICAM-1-mediated ocular inflammatory diseases. However, further studies are needed to elucidate the detailed mechanisms.

## Data Availability

Not applicable.
